# 69 Identifying Longitudinal Outcome Domains Post-Burn Injury: A Systematic Review of Validated Patient-Reported Outcome Measures

**DOI:** 10.1093/jbcr/iraf019.069

**Published:** 2025-04-01

**Authors:** Shanmuga Priya Rajagopalan, Andrea Gongora, Lorreen Agandi, Victoria Goodrich, Fasika Abreha, Isaac Obeng-Gyasi, Mark Fisher, Julie Caffrey, Carisa Cooney

**Affiliations:** Johns Hopkins Bayview Medical Center; Johns Hopkins University School of Medicine; Johns Hopkins University School of Medicine; Johns Hopkins University; Johns Hopkins University; University of California, Los Angeles; Johns Hopkins University; Johns Hopkins University School of Medicine; Johns Hopkins University School of Medicine

## Abstract

**Introduction:**

Burn injuries represent a significant public health concern. Patient-reported outcome measures (PROMs) are used to assess physical and psychological outcomes. We reviewed the literature to identify (1) commonly-used PROMs that assess long-term outcomes and (2) domains of interest not addressed by burn-specific PROMs.

**Methods:**

We searched 3 databases (PubMed, Embase, Web of Science) from 1979-July 2024. Eligible articles studied adult burn patients (≥18 years) with a follow-up period of 13-24 months using either a burn-specific or combination of burn- and non-burn-specific, validated PROMs. Six reviewers independently screened articles; three conducted quality assessments using JBI critical appraisal of bias tools. We used COVIDENCE for article screening and Microsoft Excel for data analysis.

**Results:**

Our initial search yielded 29,644 articles. Following title, abstract, and full-text screening, 7 studies containing 706 patients were eligible for inclusion. Burn injury types included thermal, electrical, and chemical. TBSA was listed as a mean of ≤50% in two (n=2) studies and >50% in two (n=2) studies, a median of 5% in one (n=1) study, and not listed in two (n=2) studies. Four (n=4, 57%) studies used a combination of validated burn and non-burn-specific PROMs (“combination studies”), such as Burn Specific Health Scale (BSHS), Hospital Anxiety and Depression Scale (HADS), and Impact of Event Scales (IES). Three (n=3) studies used only validated burn-specific questionnaires: Vancouver Scar Scale (VSS) and Patient and Observer Scar Assessment Scale (POSAS)]. Studies using only burn-specific PROMs focused on outcomes related to burn scar treatment and management. Combination studies assessed psychological and functional aspects of patient recovery such as anxiety and depression (HADS, n=1) and PTSD symptoms (IES, n=2). The most common burn-specific questionnaire used in combination studies was the BSHS (n=4). Of the three (n=3) studies that used only burn-specific questionnaires, two (n=2) used one PROM each to assess burn scars [VSS (n=1), POSAS (n=1)] and one (n=1) study used both to help address “physical restriction” and “psychological strain” of scars as well as “general scar assessment.” Substantial data heterogeneity prevented meta-analysis.

**Conclusions:**

Burn-specific PROMs primarily assess scar management, necessitating combined use of burn- and non-burn-specific PROMs to assess psychological, functional, and emotional impacts of burn injury and long-term outcomes. Burn research and patient care could benefit from creating and validating a burn-specific PROM assessing psychological and functional domains vital to the long-term well-being of burn survivors.

**Applicability of Research to Practice:**

This research provides data on burn- and non-burn-specific PROMs to assess long-term outcomes of burn survivors.

**Funding for the Study:**

N/A

